# Physics-Informed Neural Network Prediction of Methane Hydrate Flash Crystallization and Dissociation in Microfluidic Confinement

**DOI:** 10.1021/acs.energyfuels.6c01254

**Published:** 2026-07-08

**Authors:** Seth Dale, Erfan Behravesh, Amartya Singh, Alvin Li, Carolyn A. Koh, Ryan L. Hartman

**Affiliations:** † Department of Computer Science, Colorado School of Mines, Golden, Colorado 80401, United States; ‡ Department of Chemical and Biomolecular Engineering, NYU Tandon School of Engineering, Brooklyn, New York 11201, United States; § Department of Chemical & Biological Engineering, Colorado School of Mines, Golden, Colorado 80401, United States

## Abstract

Methane hydrates—ice-like crystalline solids that trap methane within a water lattice—represent both a potential energy resource and a critical factor in climate change due to their sensitivity to environmental disturbances. Modeling their formation and dissociation involves complex thermodynamic and kinetic interactions that are difficult to capture with traditional numerical methods, particularly when experimental data are limited. The challenge is further amplified when crystallization is governed by mixed transport- and growth-limited processes occurring on time scales of seconds, as is often the case for methane hydrates. This work reexamines empirical data sets for methane hydrate crystallization and dissociation through Physics-Informed Neural Networks (PINN). The PINN framework operates directly on the governing heat and mass transfer differential equations through a neural-network field representation, meaning errors introduced by the simplifications required to yield closed-form solutions or grid discretization are not admitted by our approach. The presented models efficiently solve the inverse problem. For the growth data set, the PINN lowers the in-sample velocity RMSE by 23.5% relative to the closed-form serial-resistance model of the original study (3.26 versus 4.26 μm/s) while replacing the eighteen per-pressure constants of that model with seven global physical parameters. For the dissociation data set, a single global set of kinetic parameters in place of the per-curve fits reproduces all conditions to within the experimental noise floor and recovers an apparatus-level intrinsic rate constant in agreement with the original analysis. Moreover, leave-one-out cross-validation results indicate generalizability to unseen conditions, a capability not offered by the per-condition fittings. This work establishes PINNs as a scalable and computationally efficient approach for hydrate modeling, bridging the gap between data-driven and physics-based methods. The framework offers broad potential for applications in energy production, carbon sequestration, and climate modeling.

## Graphical abstract

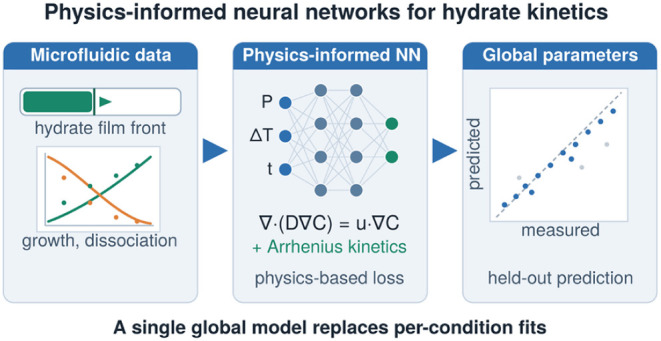

## 1 Introduction

Methane gas hydrates are crystalline solid structures where methane molecules are trapped within a lattice of water molecules under specific conditions of low temperature and high pressure.^1^ These hydrates are predominantly found in marine sediments and permafrost regions and are considered a promising alternative energy resource. Estimates indicate that global methane hydrate reserves surpass conventional natural gas deposits, positioning them as a viable energy source for the future.^2^ However, methane hydrates also have significant implications for climate change, as their destabilization due to geological or environmental disturbances can lead to the release of methane—a potent greenhouse gas—into the atmosphere.^3^

Existing hydrate growth models are often apparatus-dependent and only fit data within their own parameter space.^2^ They particularly fall short when extrapolated to low subcooling conditions, where both heat and mass transport limitations become critical.^4,5^ In this regime, the driving forces at the gas–liquid–solid interface are minuscule, and molecular diffusivity decreases with subcooling, making conventional batch reactors inadequate for isolating kinetics.^2,6^ Microfluidics provide a unique solution: their small length scales, high surface-to-volume ratios, and controlled fluid dynamics enable precise dissection of coupled heat- and mass-transfer effects under hydrate-forming conditions.^7,8^ Combined with *in situ* spectroscopic techniques, microfluidic platforms allow real-time observation of crystallization processes, uncovering transitions such as the shift from heat-transfer-limited to mixed heat- and mass-transfer-limited growth at the methane–water–hydrate interface.^7,8^ These features make microfluidics an exceptional experimental system for generating the high-quality kinetic data needed to train and validate predictive models.

Understanding and modeling these thermodynamic and kinetic mechanisms is crucial for multiple applications, including energy extraction, carbon sequestration, and subsea pipeline integrity in the oil and gas industry.^9^ Traditionally, numerical models based on phase equilibrium calculations and computational fluid dynamics (CFD) simulations have been employed to study hydrate behavior. These models solve coupled partial differential equations (PDEs) governing heat transfer, mass transport, and phase transitions.^4,10,11^ However, these models are computationally intensive—often requiring fine-resolution meshing and custom solvers—and their accuracy is strongly dependent on the availability of complete, high-quality data, which may be sparse or uncertain in many practical scenarios.^12,13^ These limitations underscore the need for alternative approaches that combine physical fidelity with data efficiency—such as Physics-Informed Neural Networks (PINNs).

PINNs have emerged as an innovative approach to addressing these challenges. First introduced by Raissi et al.,^14^ PINNs integrate machine learning techniques with physical laws by embedding governing equations as constraints within the loss function of a neural network. Unlike purely data-driven models, which may struggle with generalization, PINNs ensure physically consistent predictions even in the presence of limited experimental data. Their ability to leverage automatic differentiation and bypass numerical discretization makes them particularly advantageous for modeling complex, nonlinear phenomena in chemical and petroleum engineering.^15,16^

Recent studies have demonstrated the effectiveness of PINNs in solving multiphysics problems, including momentum transfer, heat transfer, mass transport, and reaction kinetics. For example, Ngo and Lim^17^ successfully applied PINNs to solve inverse problems and identify kinetic parameters in catalytic CO_2_ methanation within a fixed-bed reactor. Similarly, Huang et al.^18^ used an inverse-forward PINN framework for optimal membrane reactor design under nonlinear reaction-transport conditions, while Ji et al.^19^ demonstrated PINNs’ capability to accurately simulate stiff chemical kinetic systems, overcoming the significant computational hurdles posed by their diverse reaction time scales. These examples underscore the strength of PINNs in capturing nonlinear, coupled physical systems with limited data availability and high model complexity. Given their demonstrated capabilities in solving forward and inverse problems with embedded physics, PINNs present a promising alternative for predicting methane hydrate association and dissociation dynamics.

This study aims to develop a robust PINN-based framework for modeling methane hydrate behavior under varying thermodynamic conditions. Building on our previous works on hydrate kinetics in microfluidics,^20,21^ which provide both experimental data and governing equations for heat transfer and crystallization dynamics, we integrate these elements into the model architecture. By comparing PINN predictions to traditional fitting methods, this work evaluates the feasibility of PINNs as a scalable solution for hydrate-related applications in energy production and climate modeling. The foundational equations used closely capture the dynamics of methane hydrate association and dissociation and serve as the basis for model development. Methane hydrates are a particularly challenging class of crystallizations in the energy field to study, requiring cryogenic temperatures and elevated pressures. Our findings lay the groundwork for use of PINNs in other complex materials predictions in applied energy.

Physics-informed neural networks (PINNs) offer a route to a globally constrained inverse problem. By representing the latent fields (concentration, temperature, or extent of reaction) with neural networks and enforcing the governing differential equations as residuals at random collocation points, PINNs fit a single global set of physical parameters across all experimental conditions simultaneously. We apply this framework to two independent analyses of the Chen et al.^20,21^ apparatus:

1 A *growth PINN* that solves the dimensionless 1-D steady-state two-field convection-diffusion problem in the front-attached coordinate, with simultaneous mass-balance and heat-balance closures at the moving front. Seven global physics scalars replace the eighteen per-pressure constants of Chen et al.^20^
2 A *dissociation PINN* that solves a Stefan/intrinsic-kinetics coupled ODE on the remaining hydrate mole fraction. Two global physics parameters replace the sixteen per-curve parameters of Chen and Hartman.^21^

Beyond the parsimony benefit, recovering global Arrhenius activation energies provides a check against the broader hydrate-kinetics literature: the values for both directions fall within the wide range of previously reported activation energies. We also explicitly characterize the identifiability of each model, identifying degenerate ridges masked by per-condition regressions.

## 2 Background

### 2.1 Methane Hydrate Kinetics: From Per-Condition Models to Apparatus-Level Rate Constants

Methane (sI) hydrate dissociation and growth kinetics have been studied for nearly four decades, and a consistent picture has emerged of three competing rate-limiting processes: heat transfer, mass transfer, and intrinsic surface kinetics. The dominant process is dependent on the experimental apparatus, the imposed driving force, and the morphology of the hydrate film itself.

The intrinsic-kinetics framework dates to the Kim–Bishnoi rate law,^22,23^ which posits a first-order surface reaction with an Arrhenius temperature dependence:

1 $$k_{d}\left(T\right)=k_{0}\times \exp\left(\frac{E_{a}}{RT_{eq}}\right)$$

Reported activation energies include Kim et al.’s original value of approximately 78.3 kJ/mol and Clarke and Bishnoi’s refit of approximately 81 kJ/mol.^22,23^

Heat-transfer-limited models, derived by Selim and Sloan,^24^ Yousif et al.,^25^ and Hong et al.,^26^ treat dissociation as a moving-boundary Stefan problem in which conduction through a dissociated water–methane film sets the rate. The resulting expressions contain an effective thermal conductivity, a film-thickness parameter, and a power-law dependence on the temperature driving force, with empirical exponents in the range 1.5 to 2.5 commonly reported.^27^ In larger batch reactors with millimeter-to-centimeter hydrate films, this regime dominates and apparent activation energies are correspondingly biased downward by the transport rate limit.

The 2010s saw a methodological turn toward microfluidic study of hydrate kinetics, driven by the recognition that conventional batch reactors have large thermal mass and convective heat transfer that obscure the intrinsic kinetics. Microfluidic channels, with cross-sectional dimensions of order 100 μm, micrometer-scale hydrate films, conductive heat transfer with Reynolds numbers of order unity, and isothermal control to within 0.1 K, can in principle isolate the intrinsic kinetics from transport contributions. *In situ* Raman spectroscopy provides quantitative time-resolved measurement of the hydrate cage occupancy through characteristic shifts at 2915 cm^–1^ (small 5^1 2^ cages) and 2905 cm^–1^ (large 5^12^6^2^ cages).^28^

Chen et al.^20^ extended this approach to quantitative kinetic measurement. They reported planar methane-hydrate film propagation rates in a thermoelectrically cooled silicon-Pyrex microreactor at six pressures (30 to 80 bar) and seven subcoolings per pressure (1.0 to 4.0 K). They derived a serial-resistance model coupling heat transfer, mass transfer through a stagnant film ahead of the propagating front, and intrinsic crystallization kinetics, fitting three constants (k_p, k_c, h_p) at each pressure. Their dimensional analyses (Lewis number 128 to 207, Hatta number 0.5 to 0.68) demonstrated that the diffusive flux through the stagnant film contributes meaningfully—a regime not captured by purely heat-transfer-limited models.

Chen and Hartman^21^ reported methane-hydrate dissociation time series at three pressures (60.2, 70.3, 80.1 bar) and three superheats (ΔT = 0.10, 0.20, 0.30 K) above the equilibrium boundary. They derived a coupled heat-transfer + intrinsic-kinetics closed-form expression (their eq 4) and fitted two parameters per curve: the initial film thickness *l_i_* and an intrinsic rate constant *k_d,i_*. Their reported activation energy of 90.95 ± 12.15 kJ/mol emerged from a downstream linear regression of log *k_d,i_* on 1/*T_eq_* (*P*) across the nine fitted *k_d,i_* values.

Both Chen analyses are quantitatively successful at the per-condition level, but their shared methodological feature—independent per-condition parameter fits—limits what can be concluded from them. With three parameters per pressure in the growth model and two per curve in the dissociation model, condition-specific morphological variation, such as the initial film thickness, can be absorbed into the apparent kinetic parameters, leaving any subsequent Arrhenius regression sensitive to whether the absorbed variation is itself temperature-correlated. The independent recovery of an apparatus-level rate constant and activation energy from a globally constrained model is not directly addressable in this framework, nor is held-out prediction of unseen conditions: per-curve fitting necessarily assigns each curve its own parameters, leaving no quantities to evaluate on a held-out condition.

The motivation for a global inverse-problem reanalysis is therefore methodological as much as scientific. The Chen data sets^20,21^ are detailed enough to identify global apparatus-level kinetic parameters if a model can be brought to bear that demands global consistency across all conditions; that is the role of the PINN framework we adopt.

### 2.2 PINNs and the Inverse-Problem Loss-Balance Problem

PINNs^14^ use a neural-network ansatz for the latent field of a differential equation and minimize a loss that combines a data-misfit term with one or more physics-residual terms evaluated at random collocation points within the domain. For a generic PDE F­[u; θ] = 0 with unknown field u and parameters θ, the canonical PINN loss is

2 $$L=\lambda_{\mathrm{data}}\times L_{\mathrm{data}}\left(u_{NN}\right)+\lambda_{\mathrm{pde}}\times L_{\mathrm{pde}}\left(u_{NN};\theta \right)+\lambda_{\mathrm{bc}}\times L_{\mathrm{bc}}\left(u_{NN}\right)+\cdot \cdot \cdot$$

with $L_{\mathrm{data}}$ capturing the data misfit and $L_{\mathrm{pde}},\;L_{\mathrm{bc}}$ the squared physics residuals at collocation points. In forward problems *θ* is given and *u* is unknown; in inverse problems both *u* and *θ* are learned jointly, making PINNs a flexible tool for parameter identification from sparse or noisy measurements of dynamical systems. The framework has since been applied across the physical sciences, with reviews by Karniadakis et al.^16^ and Cuomo et al.^29^ covering the field’s first half-decade.

A persistent technical difficulty in PINN training is the balance among loss components. The residuals $L_{\mathrm{pde}},\;L_{\mathrm{bc}},\;L_{\mathrm{data}}$ and any additional physics constraints can differ by many orders of magnitude in numerical scale, depend on the problem’s nondimensionalization, and evolve at different rates during training. Empirical studies^30,31^ have documented severe failure modes when the weights are poorly chosen—gradient-flow pathologies, neural-tangent-kernel imbalances, and training stagnation where one residual dominates the gradient signal. Several mitigation strategies have appeared in the literature, including manual hand-tuning of relative weight, learning-rate annealing of individual loss components based on gradient-norm balancing,^30^ neural-tangent-kernel weighting,^31^ self-adaptive architectures,^32^ and causal training^33^ for time-dependent problems. These methods are increasingly sophisticated, but each adds hyperparameters of its own and most lack a clean probabilistic interpretation; the fitted relative weights, where they exist as explicit quantities, are not easily related to physical or statistical properties of the problem.

An alternative framing, originating in multitask computer vision, is Kendall and Gal’s homoscedastic-uncertainty weighting.^34,35^ For a multitask loss with *K* Gaussian heads, each head’s loss $L_{i}$ is wrapped as

3 $$L_{i,\mathrm{NLL}}=\frac{L_{i}}{2\sigma_{i}^{2}}+log\sigma_{i}$$

with *σ_i_* a learnable scalar. The log *σ_i_* term acts as a regularizer that prevents the trivial solution *σ_i_* → ∞, which would drive the first term to zero. At maximum likelihood, $\sigma_{i}^{2}$ equals the mean squared residual of its term, so the effective weight $1/\left(2\sigma_{i}^{2}\right)$ autobalances against the current residual scale during training. The formulation has a clean Bayesian interpretation as the negative log-likelihood (NLL) of a Gaussian observation model with task-dependent variance, and the fitted *σ_i_* values are directly interpretable as the model-implied noise scale on each task’s predictions.

The formulation transfers to PINN inverse problems where the tasks are the various data and physics-residual streams. The data NLL with a learnable *σ_v_* is a particularly natural identification mechanism for measurement noise. The physics-residual NLLs, with *σ* values on dimensionless normalized residuals, provide constraint-tightness diagnostics: small *σ* means the constraint is well-satisfied at convergence; large *σ* means the model has been forced to relax the constraint to fit the data. To our knowledge, no published work applies Kendall weighting uniformly to all residual streams of an inverse-PINN—most adaptive PINN weighting schemes treat the data term separately from the physics residuals or apply per-collocation-point rather than per-term weights. The application of Kendall weighting to inverse-PINN hydrate kinetics is, to our knowledge, novel. In the next section, we develop the two PINN models concretely. The framework is the same; only the governing physics and parametrization differ between the two experiments.

## 3 Methodology

Detailed here is the methodology used for both hydrate association and dissociation, outlining the experimental data, neural network architecture, loss function formulation, and computational resources. The PINN framework is designed to enhance predictive accuracy while ensuring adherence to physical principles, offering a robust tool for understanding methane hydrate behavior in microfluidic and natural systems.

### 3.1 Experimental Data

For hydrate crystallization, we leverage experimental data from the work of Chen et al.,^20^ which investigated methane hydrate crystallization kinetics in a thermoelectrically cooled microreactor. In this system, methane hydrate association was monitored under controlled subcooling temperatures (1.0–4.0 K) and pressures (30.0–80.9 bar). The experimental setup included precise temperature cycling, Raman spectroscopy for *in situ* characterization, and a high-resolution imaging system for capturing propagation rates. Key findings from this work include:

Propagation rates of methane hydrate thin films ranged from 3.1 to 196.3 μm/s, depending on the operating conditions.
Growth regimes were observed to transition from heat-transfer-dominated to mixed heat- and mass-transfer-limited kinetics at the methane–water interface.

These results provided essential inputs for developing and validating the PINN model. Chen et al.^20^ shows that two regimes are fundamental to methane hydrate crystallization: conductive heat transfer controls crystal growth, while mixed mass-transfer kinetics control film growth. The heat transfer rate (*r_h_*) is governed by energy balance at the interface and is critical for maintaining temperatures below the hydrate phase boundary. The propagation rate when conductive heat transfer controls crystal growth is expressed as:

4 $$r_{h}=\frac{dx}{dt}=\frac{h^{\prime }{\left(T_{eq}-T_{b}\right)}^{n}}{\rho_{H}\Delta H}$$

Where *h*′ is the effective heat transfer coefficient, *T_eq_* is the phase equilibrium temperature, *T_b_* is the bulk temperature, *ρ_h_* is the hydrate density, and Δ*H* is the enthalpy of crystallization. This equation emphasizes the exothermic nature of hydrate association and the role of temperature gradients in determining the propagation rate. This equation assumes steady-state propagation with dominant conductive heat transfer, isothermal operation enabled by high thermal conductivity silicon substrates, and exothermic crystallization that requires heat removal to maintain subequilibrium temperatures. However, this approach is limited by its inability to accurately predict growth at low subcooling temperatures (<4 K), reliance on apparatus-dependent parameter fitting, and failure to account for mass transfer resistances that become significant under mild subcooling conditions.

When mixed mass transfer-kinetics controls film growth, the rate (*r_c_*) is given by:

5 $$r_{c}=\frac{dx}{dt}=\left(\frac{k^{\prime }k_{c}}{k^{\prime }+k_{c}}\right)\left(\frac{M_{CH_{4}}}{\omega_{CH_{4}}\rho H}\right)C_{m}$$

where *k*′ is the apparent specific crystallization rate constant in excess water, *k_c_* is the mass transfer coefficient of methane, $M_{CH_{4}}$ is the molecular weight of methane, $\omega_{CH_{4}}$ is the weight fraction of methane in the hydrate, and *C_m_* is the methane concentration at the interface. This model captures the interplay between mass transfer resistance and intrinsic crystallization, particularly in conditions of mild subcooling and low methane solubility. This equation assumes methane as the limiting reactant due to its low aqueous solubility (0.0974 mol kg^–1^), first-order crystallization kinetics, Fickian diffusion through a stagnant film (*Δ*_*s*_ ≈ 0.13 μm), and Arrhenius temperature dependence for the intrinsic rates. The model is constrained by its validation range (30.0–80.9 bar, 1.0–4.0 K subcooling), microfluidic-specific geometric effects, and its applicability being limited to pure methane (sI) hydrate systems.

Further, for hydrate dissociation, we built on the work of Chen et al.^21^ who investigated methane hydrate dissociation kinetics using a thermoelectrically cooled microreactor combined with *in situ* Raman spectroscopy. The microreactor was designed to control pressures between 60.2 and 80.1 bar and temperatures offset from the phase equilibrium by increments as small as 0.1 K. The system’s isothermal conditions minimized external disturbances, allowing the dissociation process to be examined with high precision. Methane hydrate dissociation was characterized using the normalized intensity of the 2905 cm^–1^ Raman peak, which directly correlated with the hydrate concentration. Thin hydrate films (∼10 μm) were found to dissociate under intrinsic kinetic control, whereas thicker films exhibited a shift to heat-transfer-limited dissociation. This behavior was influenced by the temperature offset, initial hydrate film thickness, and phase equilibrium conditions. These experimental observations provided a robust data set that formed the basis for developing the PINN model.

The intrinsic kinetics of methane hydrate dissociation were derived from equilibrium reactions. The dissociation rate is expressed by the eq 6:

6 $$\frac{dn_{H}}{dt}=-k_{d}n_{H}+k_{b}\times C_{m}\times C_{W}^{5.75}$$

where *k*_*d*_ is the dissociation rate constant, *k_b_* is the crystallization rate constant, *n_H_* is the number of moles of dissociated hydrates, *C_m_* and *C_W_* are the concentrations of methane and water, respectively. This equation indicates that the dissociation rate is influenced not only by the decomposition of methane hydrate but also by its reformation under certain conditions close to the phase boundary. The equilibrium constant *K_eq_* relates these processes and can be used to describe the overall dissociation behavior under various thermal and concentration conditions. The detailed understanding of these kinetics helps in the accurate modeling of methane hydrate behavior in microfluidic systems.

### 3.2 Growth PINN

The growth PINN (Figure 1) solves the steady-state mass-transport problem in the moving-front frame, with the dimensionless front-attached coordinate ξ ∈ [0,1] running from the dissociation/growth front (ξ = 0) to the bulk-liquid boundary (ξ = 1). Two multilayer perceptrons (MLPs) with four hidden layers of 64 units, tanh activations, and Glorot-normal weight initialization represent: (1) the dissolved-methane concentration *C*(*P*, Δ*T*, ξ) (mol/m^3^) with output scaled by C̃ = 2500 mol/m^3^ and (2) the temperature *T*(*P*, Δ*T*, ξ) (K) parametrized as *T*(ξ) = *T_b_* +(1 – ξ) × (*T*(0) – *T_b_*) with *T*(0) bounded to [*T_b_*, *T_eq_*] via a tanh squash.

**Figure 1:**
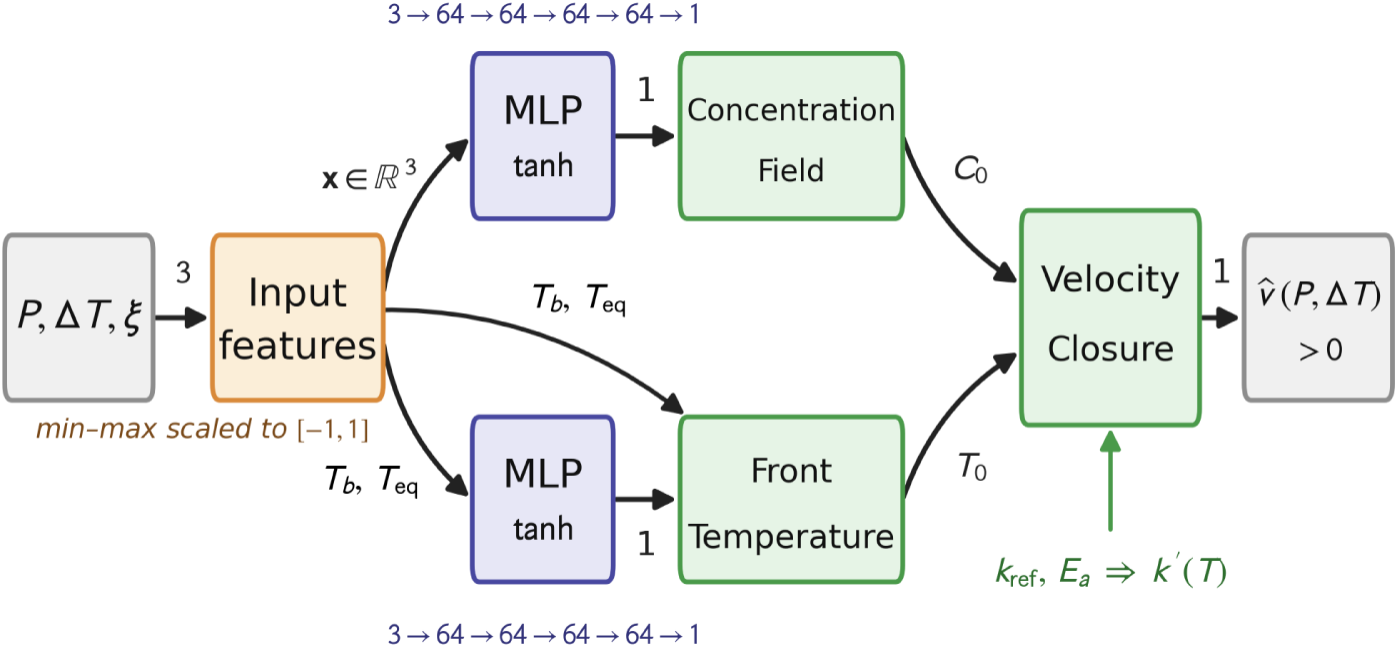
Architecture of the growth PINN. The inputs (P, ΔT, ξ), min-max scaled to [−1, 1], feed two multilayer perceptrons: a *C*-network giving the dissolved-methane concentration field *C* = 2500 × *net_C_*, and a *T*-network whose output is mapped to a hard-bounded front temperature *T*_0_ ∈ [*T_b_*,*T_eq_*].

The governing PDE is convection-diffusion of dissolved methane:

7 $$\frac{\partial }{\partial \xi }\left[D\left(T\left(\xi \right)\right)\frac{\partial C}{\partial \xi }\right]+u_{\mathrm{conv}}\delta \left(P\right)\frac{\partial C}{\partial \xi }=0$$

with *u_conv_* = (*ρ_H_*/*ρ_W_*) × *v* ≈ 0.92 and *v* is the volume-contraction convection velocity arising from the solid–liquid density mismatch as hydrate is consumed.

The boundary conditions are:

(1) Bulk side (ξ = 1): *C*(*P*, Δ*T*, 1) = *C_m_* (*P*) and (2) Front side (ξ = 0): simultaneous satisfaction of mass balance, $\frac{D\left(T\left(0\right)\right)\left(\frac{\partial C}{\partial \xi }\right){|}_{0}}{\delta \left(P\right)}+u_{\mathrm{conv}}C\left(0\right)-k^{\prime }\left(T\left(0\right)\right)C\left(0\right)=0$ , and heat balance $v\rho_{H}\Delta H=h_{\mathrm{eff}}\left(P\right){\left(T\left(0\right)-T_{b}\right)}^{n_{\mathrm{heat}}}$ with the Arrhenius rate constant:

8 $$k^{\prime }\left(T\right)=k_{\mathrm{ref}}exp\left[-\frac{E_{a}}{R}\times \left(\frac{1}{T}-\frac{1}{T_{\mathrm{ref}}}\right)\right]$$

and *T_ref_* = 273.15 K. The velocity is read out from the kinetic mass-balance closure as the following:

9 $$v=k^{\prime }\left(T\left(0\right)\right)C\left(0\right)\frac{M_{{\mathrm{CH}}_{4}}}{\omega \rho_{H}}$$

Two empirical power laws capture the dependence of the transport coefficients on pressure:

10 $$h_{\mathrm{eff}}\left(P\right)=h_{\mathrm{eff},\mathrm{ref}}{\left(\frac{P}{P_{\mathrm{ref}}}\right)}^{h_{\alpha }}$$

and

11 $$\delta \left(P\right)=\delta_{ref}{\left(\frac{P}{P_{\mathrm{ref}}}\right)}^{d_{\beta }}$$

with *P*_ref_ = 55 bar (the arithmetic midpoint of the pressure range).

This model has 12 learnable parameters: 7 physical scalars (*k*_ref_, *E_a_*, *h*_eff,ref_, *n*_heat_, *δ*_ref_, *h_α_*, *d_β_*) plus 5 Kendall noise scales (σ_v_, σ_pde_, σ_bc_, σ_fmass_, σ_fheat_). All positive-constrained scalars are log-parametrized so that the optimizer updates correspond to relative rather than absolute changes.

The total loss is given by the following:

12 $$L_{\mathrm{total}}=\underset{i\in \left\{\mathrm{pde},\mathrm{bc},\mathrm{fmass},\mathrm{fheat},\mathrm{data}\right\}}{\sum }\frac{L_{i}}{2\sigma_{i}^{2}+\log\sigma_{i}}+\lambda_{n}{\left(n_{\mathrm{heat}}-n_{\mathrm{anchor}}\right)}^{2}+\lambda_{\mathrm{mono}}L_{\mathrm{mono}}$$

The five Gaussian residual terms are automatically balanced by the learnable *σ_i_*. A weak Gaussian anchor on *n*_heat_ with λ*_n_* = 1 is included because, as we show in later sections, (*h*_eff_, *n*_heat_) admits a degenerate identification ridge; the anchor breaks the degeneracy without biasing the remaining parameters. The monotonicity penalty $L_{\mathrm{mono}}$ is a one-sided penalty on $\frac{dv}{dP}$ at fixed Δ*T* = 2.5 K with λ_mono_ = 1 × 10^4^, enforcing the physically expected monotonic increase of velocity with pressure.

### 3.3 Dissociation PINN

The dissociation PINN (Figure 2) tracks the remaining hydrate mole fraction $\eta \left(t,\;P,\;\Delta T\right)=\frac{n_{H}}{n_{0}}$ with η­(0, *P*, Δ*T*) = 1. Eliminating the front temperature from the coupled Stefan energy balance and the Kim–Bishnoi rate law yields

13 $$\frac{d\eta }{dt}=-\frac{k_{d}\eta }{1+k_{d}\alpha \eta \left(1-\eta \right)}$$

14 $$k_{d}\left(P\right)=k_{0,\mathrm{ref}}\exp\left(-\frac{E_{a}}{R}\left(\frac{1}{T_{eq}\left(P\right)}-\frac{1}{T_{\mathrm{ref}}}\right)\right)$$

15 $$\alpha \left(\Delta T\right)=\rho_{H}\Delta H\frac{l^{2}}{\left(\kappa \Delta T\right)}$$

with *T_ref_* = 282.95 K and *κ* = 0.47 W/m/K. At *η* → 1 (early time), the heat-transport factor η­(1 – *η*) vanishes and $\frac{d\eta }{dt}\to -k_{d}\eta$ is pure Arrhenius—the regime that provides clean activation-energy leverage across pressures. The peak heat-transport load is at *η*= 0.5, where the rate is slowed by the factor (1 + *k_d_α*/4).

**Figure 2:**
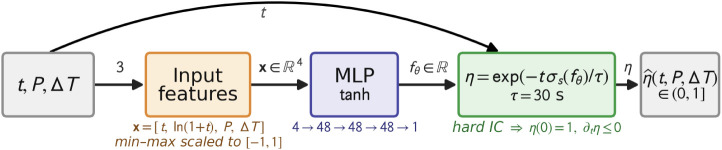
Architecture of the dissociation PINN. The inputs (*t, P, ΔT*) are expanded to the feature vector *x* = [*t*, log­(1 + *t*), *P*, Δ*T*], min-max scaled to [−1, 1], and passed to a single multilayer perceptron (4 → 48 → 48 → 48 → 1, tanh). The scalar output *f*_*θ*_ is wrapped in the hard initial-condition form η = exp­(−*t* × softplus­(*f*_*θ*_)/*τ*)) with *τ* = 30 s, which enforces *η*(0) = 1 and a monotonically nonincreasing *η*(*t*) ∈ (0,1]; the network thus predicts the remaining hydrate fraction η­(*t*, *P*, Δ*T*).

The dissociation length *l* = 7.925 μm is fixed at the apparatus mean of Chen–Hartman^21^ Table 1, excluding the outlier condition. Pinning *l* to a literature-derived prior rather than learning it eliminates an (*l*, *E_a_*) degeneracy revealed by an earlier free-*l* version and isolates the question of which kinetic parameters the data set identifies with fixed apparatus film thickness. Moreover, *l* is an unmeasured initial condition in the reference empirical study, which obstructs any potential analysis of learned *l* values.

**Table 1:** Parameter Estimates and Variance Decomposition across Random Seeds and Cross-Validation Folds^a^

Parameter	Units	Mean	*σ*_seed_	*σ*_fold_	*σ*_total_	Ratio
*k*_ref_	10^–3^ m/s	2.97	0.04	0.09	0.10	0.42
*E*_a_	kJ/mol	58.22	0.95	4.64	4.72	0.20
*h*_eff_	10^3^ W m^–2^ K^– n^	32.47	6.82	12.92	14.78	0.53
*n*_heat_	—	2.50	3.4 × 10^–3^	5.5 × 10^–3^	6.0 × 10^–3^	0.63
δ	μm	1.63	0.07	0.38	0.38	0.20
*h*_α_	—	0.60	0.69	0.74	0.9	0.94
*d*_β_	—	–0.56	0.03	0.07	0.07	0.48
σ_*v*_	μm/s	3.12	0.25	0.18	0.30	1.34

a σ_seed_ and σ_fold_ are the within-seed and within-fold standard deviations; σ_total_ = (σ_seed_² + σ_fold_²)^1/2^. ratio = σ_see_/σ_fold_.

The architecture is a single tanh-activated MLP of three hidden layers of 48 units with inputs (*t*, log­(*t* + 1), *P*, Δ*T*) each linearly mapped to [−1, 1] and outputs a raw scalar, wrapped in a hard initial-condition form

16 $$\eta \left(t,\;P,\;\Delta T\right)=\exp\left(-\frac{t}{t_{\mathrm{scale}}}\mathrm{softplus}\left({\mathrm{NN}}_{\phi }\left(t,\;P,\;\Delta T\right)\right)\right)$$

with *t_scale_* = 30 s. This guarantees *η*(0) = 1 exactly, *η* ∈ (0, 1]­for all *t* ≥ 0, and *η* monotonically nonincreasing in *t*; a separate monotonicity penalty is not required.

This model has four learnable parameters: two physical scalars (*k*_0,ref_, *E_a_*) plus two Kendall noise scales (*σ*_data_, *σ*_ode_). The logarithm of *E_a_* is clipped to log­(40 kJ/mol) ≤ log *E_a_* ≤ log­(159 kJ/mol), the physically plausible range for hydrate dissociation activation energies. The logarithm of *σ* is clipped to [−8, 2] to prevent *σ*-collapse.

Three Kendall-weighted residual streams compose the loss function:

17 $$L_{\mathrm{data}}\left(\phi ,\;s\right)=\mathrm{mean}{\left(\eta_{NN}-\eta_{\mathrm{obs}}\right)}^{2},\;L_{\mathrm{ode}}\left(\phi ,s\right)=\mathrm{mean}{\left(\frac{d\eta_{NN}}{dt}-f\left(\eta_{NN},\;P,\;\Delta T;\;s\right)\right)}^{2}$$

18 $$L_{\mathrm{total}}=\frac{L_{\mathrm{data}}}{2\sigma_{\mathrm{data}}^{2}}+\log\sigma_{\mathrm{data}}+\frac{L_{\mathrm{ode}}}{2\sigma_{ode}^{2}}+\log\sigma_{\mathrm{ode}}$$

ODE residual collocation points are sampled log-uniformly in *t* ∈ [10^–2^ s, 200 s], placing approximately 25% of points in each log-decade. This concentrates samples in the early time regime where η ≈ 1 and the heat-transport correction vanishes—the clearest Arrhenius signal across pressures.

### 3.4 Optimization and Architecture

Both PINNs are implemented in JAX with Optax. Network weights are optimized with AdamW and global scalars with Adam. The former uses weight decay as regularization, whereas standard Adam suffices for the latter—the log-parametrization already addresses parameter bias. Learning rates follow a standard cosine decay from 1 × 10^–3^ for dissociation and 5 × 10^–4^ for growth over the training horizon, with the scalar learning rate set higher (3 × 10^–3^ and 1 × 10^–3^, respectively) than the network learning rate to let the global parameters respond rapidly to gradient information. The growth PINN trains for 25,000 steps per fit with 2048 PDE collocation points and 512 boundary collocation points per step; the dissociation PINN trains for 25,000 steps with 2048 ODE collocation points per step. Gradient norms are clipped at 1.0 in the dissociation PINN. Training of each fold takes approximately 23 s for the growth PINN and approximately 5 s for the dissociation PINN on a single NVIDIA RTX 4090 GPU with 24 GB of VRAM.

### 3.5 Cross-Validation Protocol

For the growth PINN, we perform a leave-one-pressure-out (LOPO) cross-validation. This manifests as six folds, each holding out all 7 data points for one pressure and training on the remaining 35 points. Results are averaged over five random seeds, yielding 30 folds in total.

For the dissociation PINN, we perform leave-one-condition-out (LOCO) cross-validation. Eight folds are created, each holding out one (*P*, Δ*T*) condition of approximately 21 time samples and training on the remaining seven. Results are averaged over five random seeds, giving 40 folds in total.

For each scalar we report two complementary standard deviations across the multiseed cross-validation: within-seed *σ*, the seed-averaged standard deviation across folds, reflects fold-induced parameter variation and is the natural identifiability diagnostic; within-fold *σ*, the fold-averaged standard deviation across seeds, reflects optimizer noise. A within-seed *σ* to within-fold *σ* ratio much greater than unity signals real fold-induced variation; a ratio near unity signals that the spread is dominated by optimizer noise and a multiseed average should be reported as the headline value.

## 4 Results and Discussion

### 4.1 Growth PINN

For an in-sample fit across five fixed random seeds, the growth PINN reached a root-mean-square velocity error of approximately 3.26 ± 0.17 μm/s, compared with 4.26 μm/s for the Chen et al.^20^ fit, a 23.5% reduction.

Figures 3 and Figure 4 show the cross-validation performance. Across five seeds the PINN achieves an overall LOPO RMSE of 5.40 ± 0.31 μm/s. Per-fold values are 3.49 ± 0.20 (30 bar), 4.31 ± 0.53 (40 bar), 6.36 ± 0.14 (50 bar), 6.08 ± 0.08 (60 bar), 5.59 ± 0.54 (70 bar), and 8.36 ± 1.28 (80 bar). The worst-performing fold (80 bar) corresponds to the upper extremum of the pressure design and shows the largest seed-to-seed variation, consistent with reduced extrapolation reliability at the data set edge. The held-out RMSE is approximately 1.7 times the in-sample value, a typical generalization gap for a small mechanistic data set.

**Figure 3:**
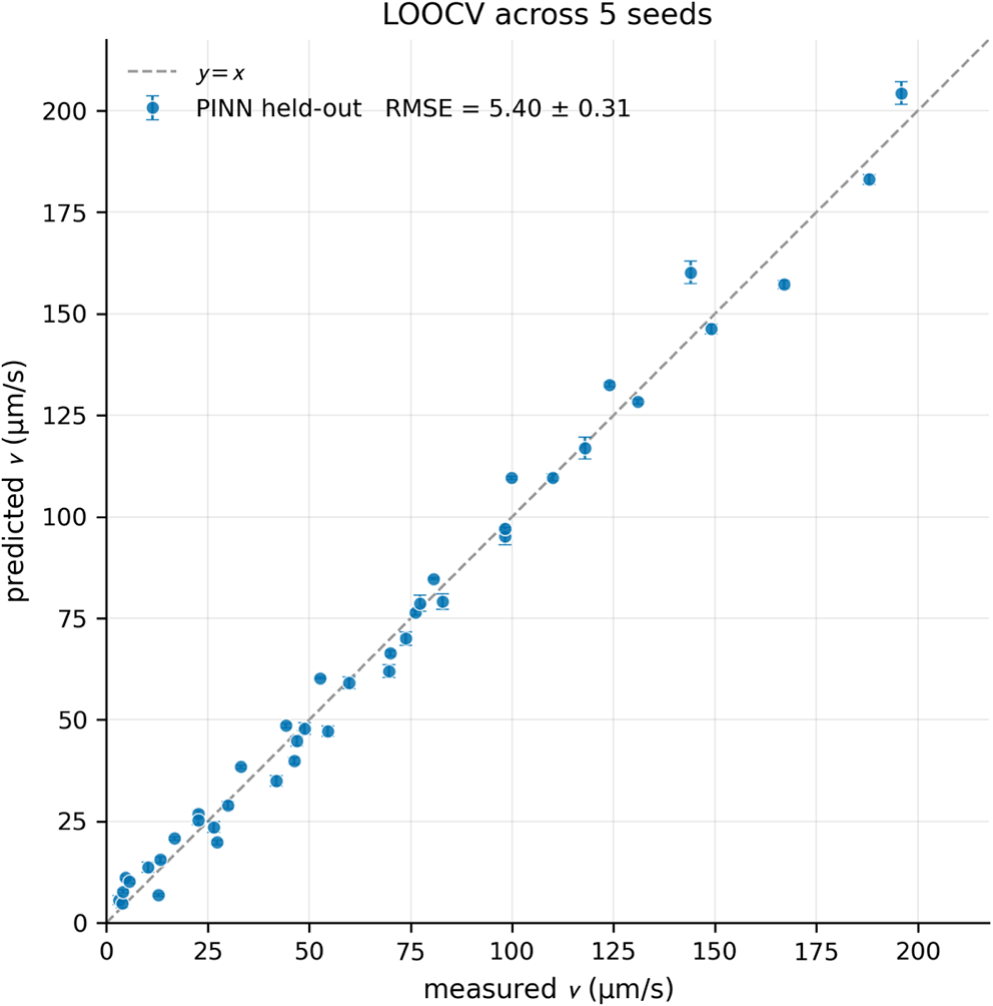
Leave-one-pressure-out (LOPO) cross-validation parity plot for the growth PINN: predicted versus measured front-propagation velocity for the held-out pressures, pooled across five random seeds. Error bars denote the seed-to-seed standard deviation and the dashed line is the 1:1 reference. The mean held-out RMSE is 5.40 ± 0.31 μm/s.

**Figure 4:**
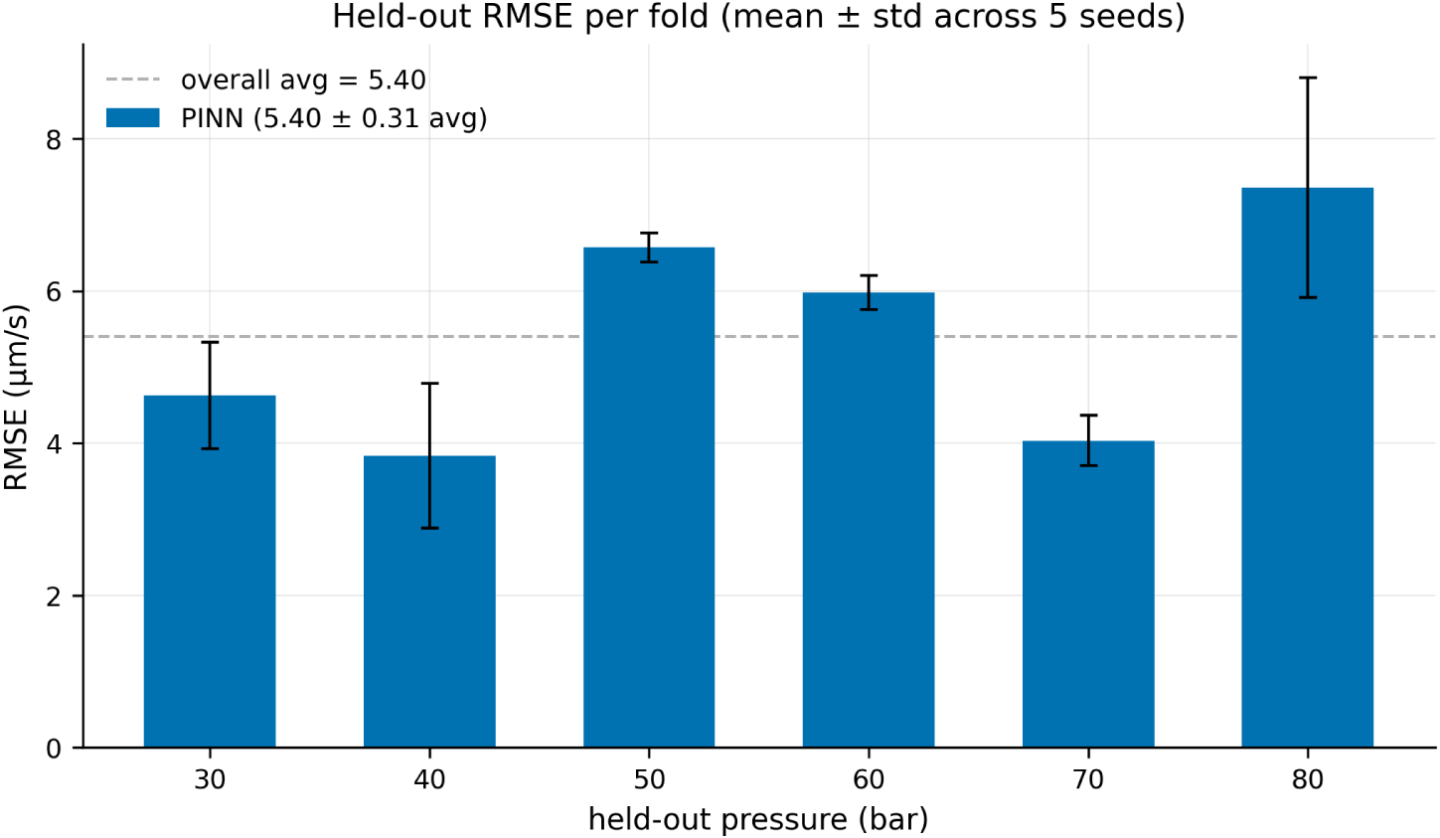
Held-out RMSE of the growth PINN for each leave-one-pressure-out fold (mean ± standard deviation across five seeds). The dashed line marks the overall average of 5.40 μm/s; the 80 bar fold at the upper edge of the pressure design shows the largest error and the largest seed-to-seed spread.

Figure 5 shows the recovered global scalars across the LOPO cross-validation. The Arrhenius and mass-transport parameters are recovered consistently across folds, as shown in Table 1.

**Figure 5:**
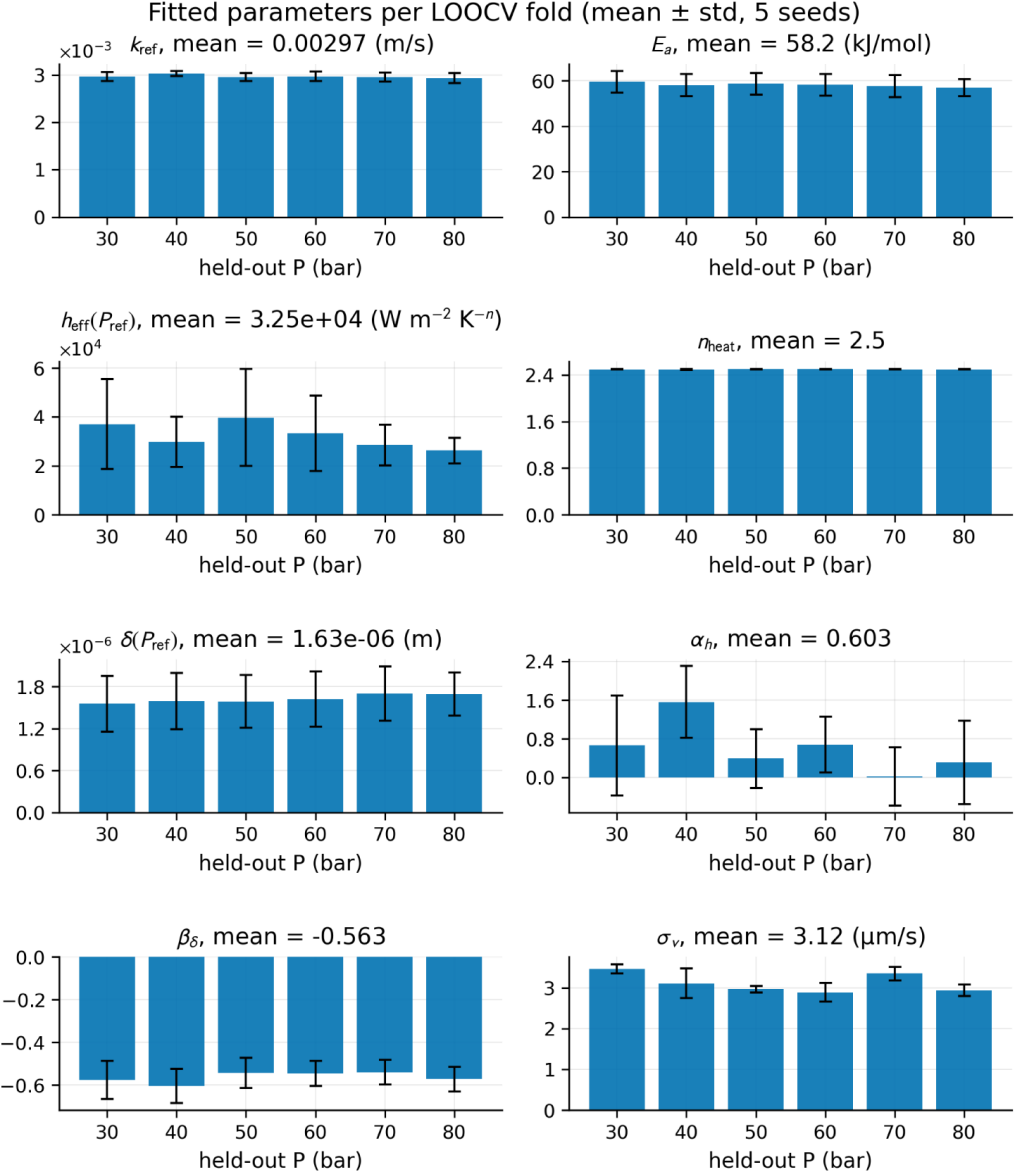
Recovered global parameters of the growth PINN across the six leave-one-pressure-out folds (mean ± standard deviation over five seeds). The Arrhenius and mass-transport parameters (*k*_ref_, *E_a_, δ*, *d*_*β*_) are stable across folds, whereas the heat-transport coefficient term *h*_*α*_ is poorly constrained.

The recovered activation energy *E_a_* is within the 40–60 kJ/mol range established in Mullin,^27^ and close to Chen et al.’s^20^ reported 64.83 kJ/mol, providing a consistency check on the inverse problem: a global PINN fit and a per-pressure Arrhenius regression agree to within fold-to-fold scatter. The mass-transport length *δ* = 1.63 ± 0.07 μm at *P*_ref_ combines with *d*_*β*_ = −0.56 ± 0.03 to give a roughly 40% reduction in *δ* across the pressure range—consistent with thinner concentration boundary layers at higher pressure and higher dissolved methane flux to the front. This dependence is invisible to Chen et al.’s^20^ per-pressure formulation, which lumps *δ* into an effective rate constant *k*_*c*_ without separating diffusive and crystallization contributions.

The variance ratio exceeds unity for *σ_v_*, meaning the model-implied measurement noise floor may vary with held-out pressure, plausibly because the underlying experimental noise model is itself condition-dependent. The other parameter ratios fall in the 0.2–0.94 range, indicating that for those parameters the fold-to-fold spread is on the same order as seed noise. We therefore report fold means as headline values, with seed-fold combined standard deviations as the natural uncertainty.

The growth heat-balance closure

19 $$v\times \rho_{H}\times \Delta H=h_{\mathrm{eff}}\left(P\right)\times {\left(T\left(0\right)-T_{b}\right)}^{n_{\mathrm{heat}}}$$

is a two-parameter family along an identification ridge: with no measurements of the front temperature T(0), the data constrain only the joint quantity *h*_eff_Δ*T* ^n^ at characteristic operating Δ*T*.

Figure 6 shows the consequences: pinning *n*_heat_ at values from 0.5 to 2.5 changes the cross-validation RMSE by approximately 8% (a 0.45 μm/s spread) and the recovered Arrhenius and mass-transport parameters by 1 to 11%. The Kendall noise scales for the physics constraints remain essentially flat across the sweep—σ_fheat_ varies by under 10%—confirming that the model satisfies each constraint equally well at every *n*_anchor_ value. This flatness signals a degenerate ridge: *h*_eff_ slides to compensate for the imposed *n*_heat_ without straining any single constraint, rather than trading fidelity between competing terms. We adopt *n*_heat_ = 2.5 for the headline configuration solely for direct comparability with Chen et al.,^20^ who used the same value as an empirical exponent in the heat-transport resistance. The choice does not materially affect any of the other recovered parameters.

**Figure 6:**
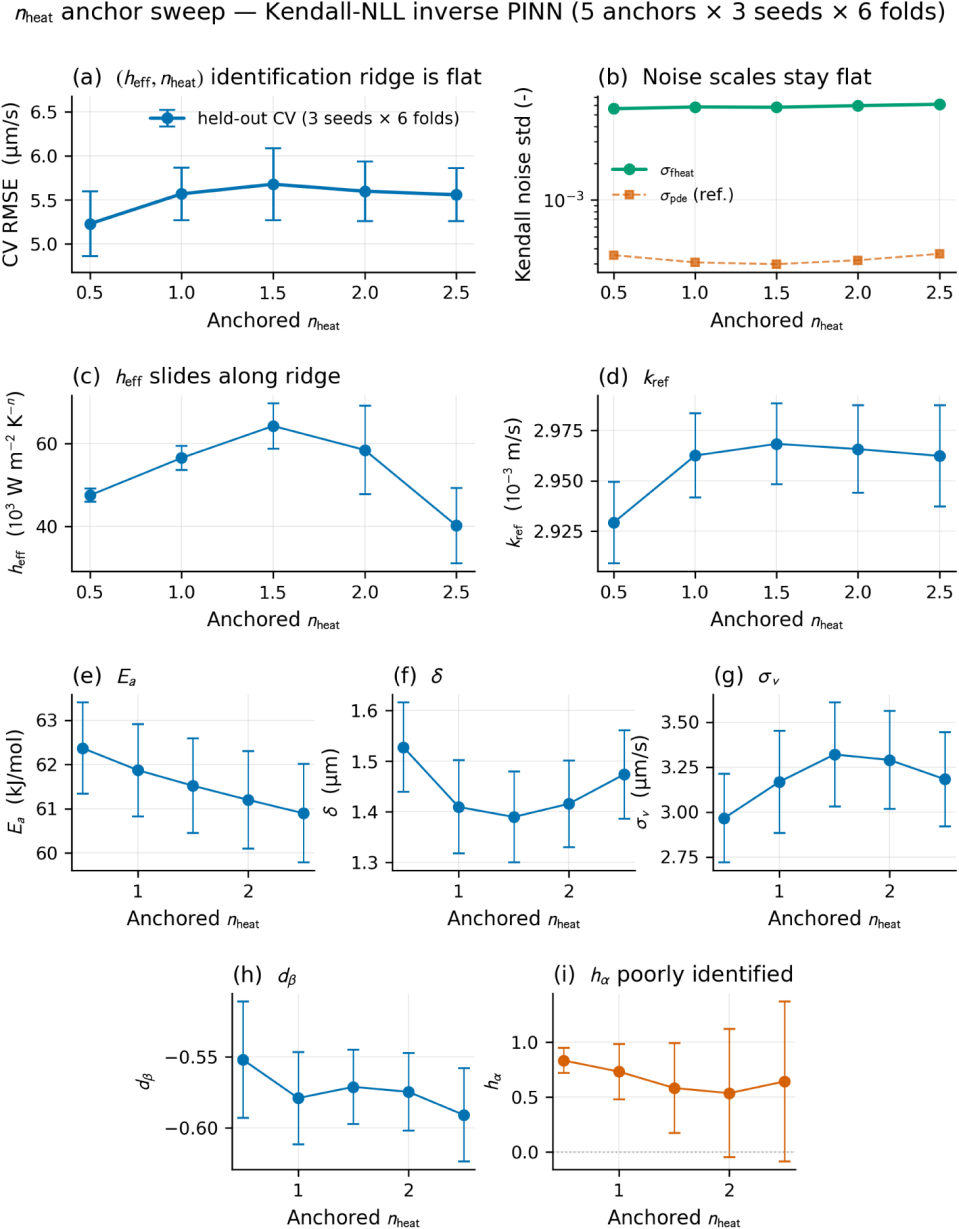
*n*_heat_ anchor sweep for the growth PINN (five anchor values × three seeds × six folds). (a) The held-out cross-validation RMSE is nearly flat along the (*h*_eff_, *n*_heat_) identification ridge and (b) the Kendall physics-residual noise scales remain flat, the signature of a genuinely degenerate ridge; (c–i) the recovered parameters versus the anchored *n*_heat_ show that *h*_eff_ adjusts to compensate for the imposed exponent while *k*_ref_, *E_a_*, *δ* vary only weakly.

The implication for hydrate kinetics is methodological rather than scientific: in data sets that constrain only the bulk-fluid temperature *T*_*b*_ and do not directly measure the front temperature, the heat-balance exponent *n*_heat_ cannot be identified independently from the heat-transport coefficient *h*_eff_. Future experiments that directly probe *T*(0) using on-front temperature reporters or higher-resolution thermometry, for instance, could break this degeneracy.

### 4.2 Dissociation PINN

Figure 7 shows the dissociation PINN in-sample fit across the eight retained conditions. Each panel overlays the experimental Raman-derived *η*(t) (points), Chen–Hartman^21^ eq 4 evaluated with the per-curve (*l_i_*, *k_di_*) values from their Table 1 (dashed), and the PINN’s global-fit prediction with *k*_0,ref_, *E_a_*, and the fixed apparatus-mean *l* = 7.925 μm (solid). The PINN’s flexible neural field reproduces all eight curves to a per-condition in-sample RMSE of 0.0005–0.0023, well below both the experimental noise and Chen–Hartman’s^21^ per-condition RMSE of 0.022–0.111. Because the PINN in-sample residual sits below the measurement-noise floor, this tightness reflects the flexibility of the field representation and is not, on its own, evidence of a superior kinetic model; the physically meaningful comparison is the held-out cross-validation (Figures 8–10). The in-sample fit establishes that the PINN reproduces the full design with only two global physics parameters constraining the kinetics across all eight panels, versus sixteen free per-curve parameters in the reference fit.

**Figure 7:**
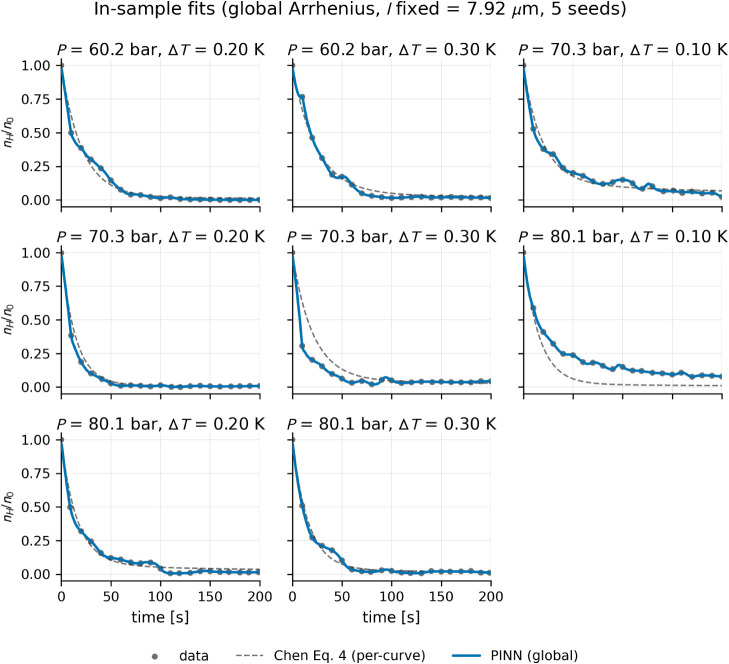
In-sample fits of the dissociation PINN for the eight retained conditions (film thickness *l* fixed at 7.925 μm, five seeds). Each panel overlays the Raman-derived hydrate fraction *n*_H_/*n*_0_ (points), the per-curve closed-form fit of Chen–Hartman^21^ (their eq 4, dashed), and the global PINN prediction (solid).

**Figure 8:**
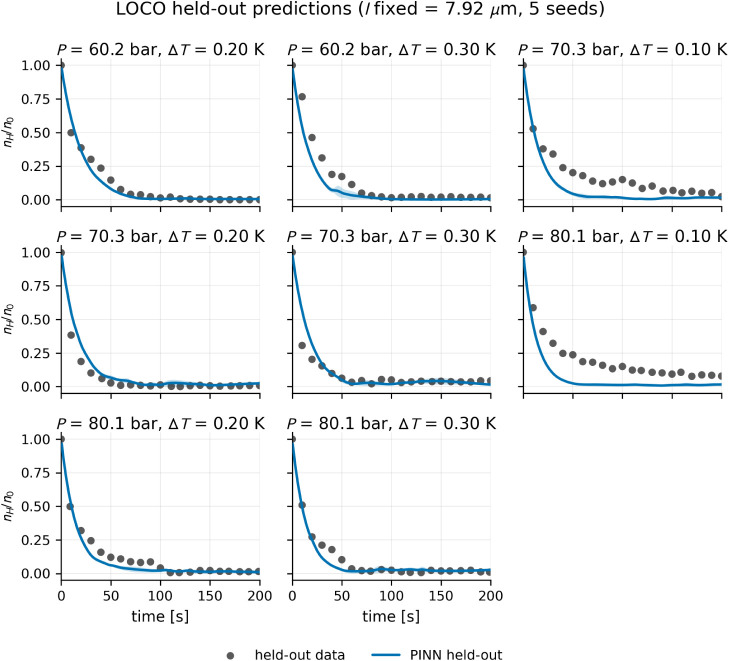
Leave-one-condition-out (LOCO) held-out predictions of the dissociation PINN ( l fixed at 7.925 μm, five seeds). Each panel shows the model prediction (line, with shaded seed band) for the (*P*, Δ*T*) condition withheld from training, compared against the held-out data (points).

Figure 8 shows the leave-one-condition-out (LOCO) held-out predictions; each panel corresponds to one fold and shows the network’s prediction on the left-out condition, with the recovered per-fold parameters summarized in Table 2 and Figure 9.

**Figure 9:**
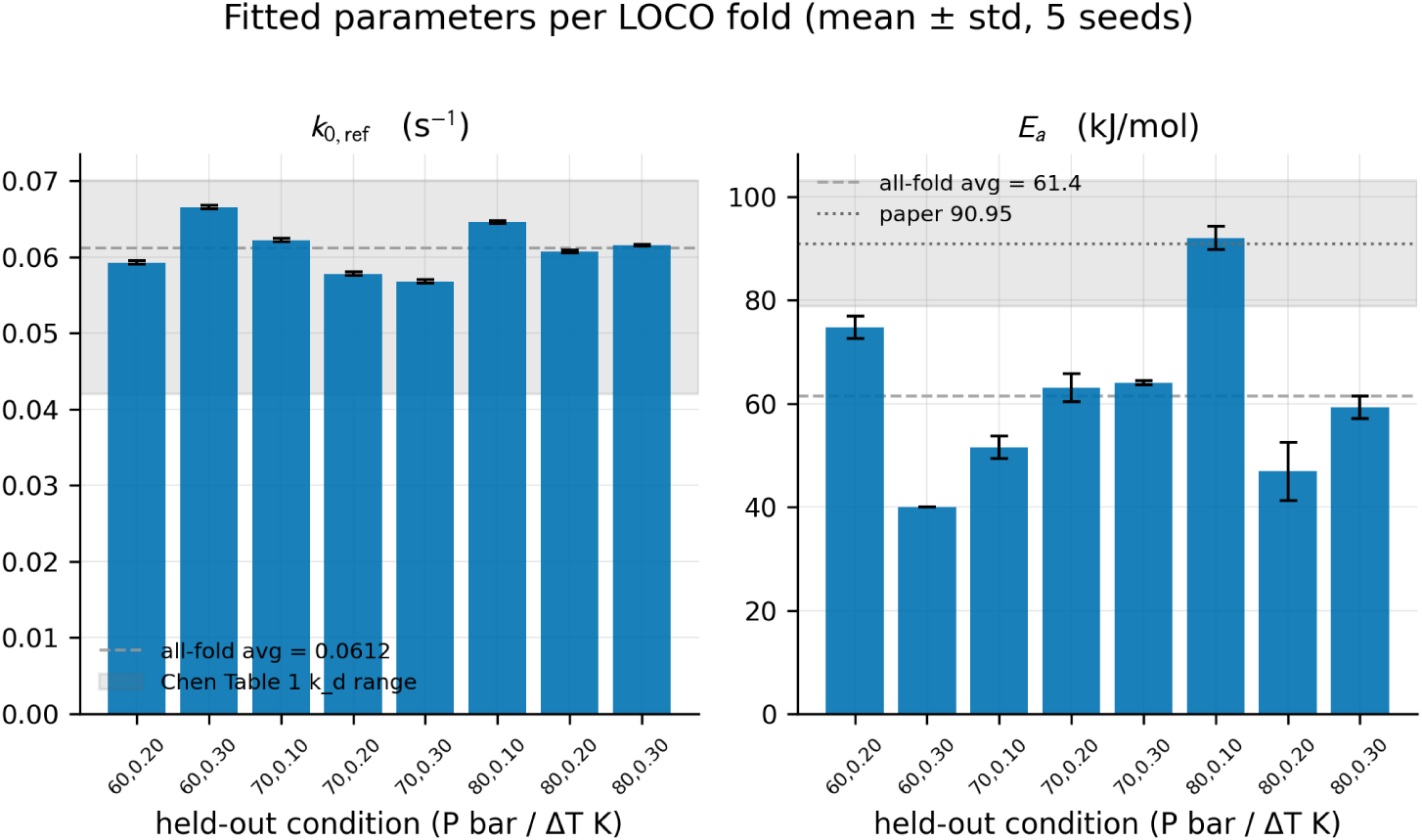
Recovered dissociation parameters for each leave-one-condition-out fold (mean ± standard deviation over five seeds). Left: pre-exponential rate constant *k*_0,ref_, with the all-fold average (0.0612 s^–1^, dashed) and the range of Chen–Hartman’s^21^ per-curve *k*_*d*_ values (shaded band). Right: activation energy *E_a_*, with the all-fold average (61.4 kJ/mol, dashed) and the per-condition reference regression value of 90.95 kJ/mol (dotted).

**Table 2:** Parameter Estimates and Variance Decomposition across Random Seeds and Cross-Validation Folds^a^

Parameter	Units	Mean	*σ*_seed_	*σ*_fold_	*σ*_total_	Ratio
*k*_0,ref_	s^–1^	6.12 × 10^–2^	3.10 × 10^–3^	2.00 × 10^–4^	3.10 × 10^–3^	15.09
*E*_a_	kJ/mol	61.44	15.49	2.18	15.63	7.10

a σ_seed_ and σ_fold_ are the within-seed and within-fold standarddeviations; σ_total_ = (σ_seed_² + σ_fold_²)­1/2. Ratio = σ_seed_/σ_fold_.

The PINN’s recovered Arrhenius prefactor is *k*_0,ref_ = 0.0612 ± 0.0001*s* ^–1^ across random seeds (0.0612 ± 0.0031 across LOCO folds), expressed as the intrinsic rate constant at the centroid reference temperature *T*_ref_ = 282.95 K. It lies within the range of Chen–Hartman’s^21^ eight per-curve *k*_*d*_ values (0.042–0.070 s^–1^) and approximately 8% above their arithmetic mean (0.0569 s^–1^). The notable feature is the stability of *k*_0,ref_, indicating that the global model robustly identifies the apparatus-level intrinsic rate constant rather than absorbing per-condition idiosyncrasies, even though its single Arrhenius prefactor must simultaneously fit all eight conditions through its *T*_eq_(*P*) dependence, as shown in Figure 9.

The activation energy *E_a_* = 56.1 ± 0.8 kJ/mol from the all-data fit (61.4 kJ/mol averaged across LOCO folds) is lower than Chen–Hartman’s^21^ regression value of 90.95 ± 12.15 kJ/mol by roughly 30–35 kJ/mol. Given the fold-to-fold identifiability spread of ±15.5 kJ/mol, this is an approximately 1.5–2σ difference, and both values lie within experimental error of literature methane hydrate dissociation activation energies. The discrepancy traces to a structural difference between the two analyses, not to a model failure. Chen–Hartman’s^21^ two-stage analysis fits (*l_i_*, *k_di_*) per curve and then regresses log *k*_*d*_ on 1/*T*_*eq*_; per-curve *l*_*i*_ is free to absorb morphological variation that may correlate with pressure, and any such variation yields an inflated Arrhenius slope at the regression step. The PINN’s analysis fixes *l* to the apparatus mean for all conditions, attributing any residual morphological variation across (*P*, Δ*T*) explicitly to the kinetic term; if the true condition-specific film thickness has a real pressure correlation, this attribution deflates the apparent *E_a_*. This data set has limited Arrhenius leverage (three pressures across a 2.9 K span of *T*_*eq*_, no replicates) and the recovered *E*_*a*_ is sensitive to how morphological variation is partitioned between *l* and *k*_*d*_.

Figure 10 shows the dissociation PINN’s per-fold held-out RMSE. Mean held-out RMSE is 0.071 across folds. Five of the eight folds achieve RMSE in the 0.04 to 0.07 range, on the order of the per-curve in-sample residuals of Chen–Hartman’s^21^ fit (overall mean 0.049)—a notable result given that these are genuine predictions of entirely unseen conditions rather than in-sample fits. The remaining three folds are higher: the two lowest-superheat conditions (Δ*T*= 0.10 K, RMSE 0.10 and 0.13) and the low-pressure, high-superheat corner (P = 60.2 bar, Δ*T* = 0.30 K, RMSE 0.08). The Δ*T* = 0.10 K conditions exhibit slower late-time decay than the global model with apparatus-mean *l* predicts; if a per-curve free *l*_*i*_ were permitted, the per-curve fit would absorb this into a larger fitted thickness at low Δ*T*, but the global PINN cannot perform this by construction. This is the natural cost of removing per-condition free parameters: predictive RMSE at the data set’s design corners is degraded relative to a per-condition fit, in exchange for global parameter transferability that per-condition fitting cannot provide.

**Figure 10:**
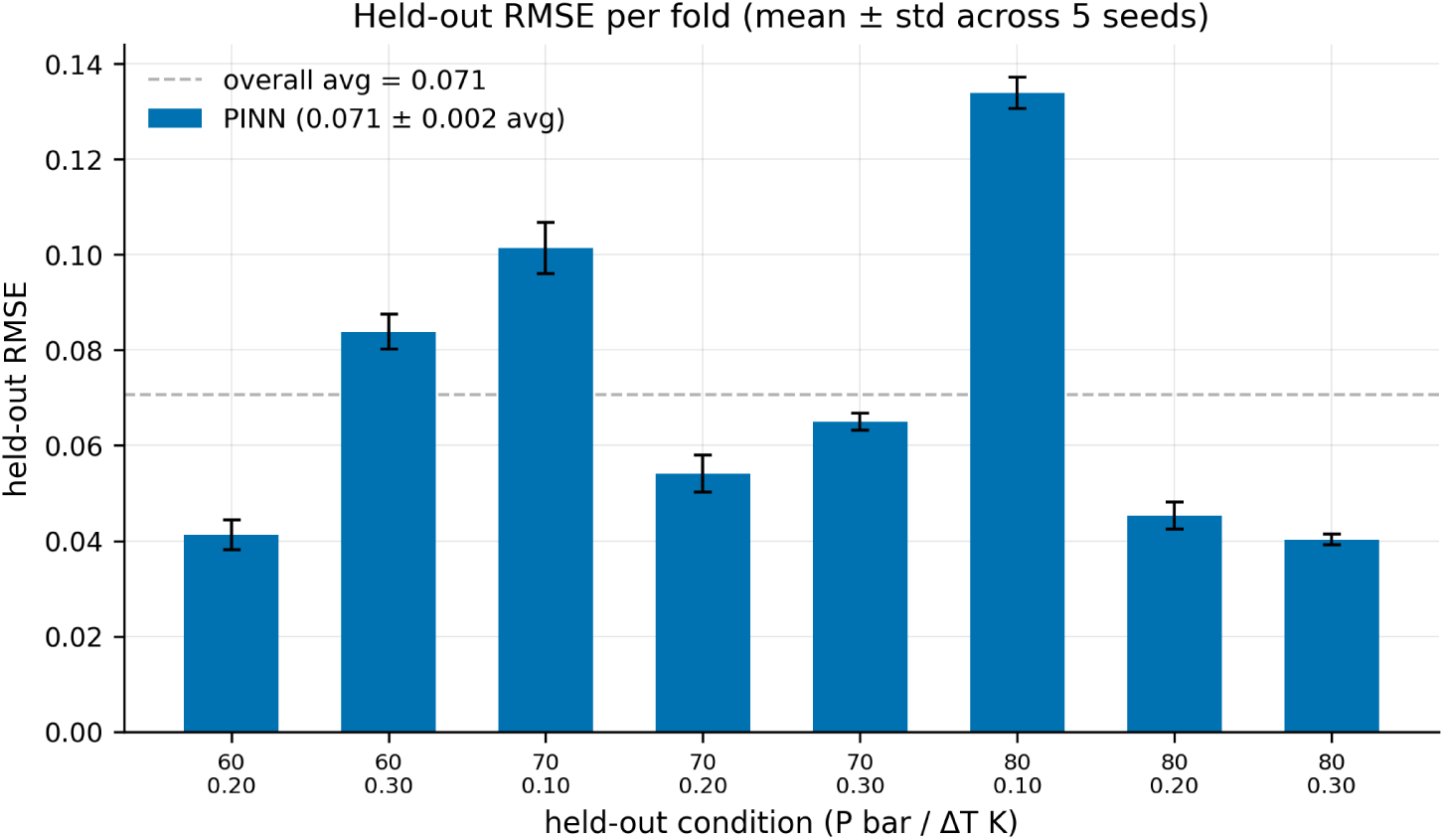
Held-out RMSE of the dissociation PINN for each leave-one-condition-out fold (mean ± standard deviation across five seeds). The dashed line marks the overall average of 0.071; the two lowest-superheat (Δ*T* = 0.10 K) conditions show the largest held-out error.

We emphasize that leave-one-condition-out cross-validation is a generalization protocol that per-curve fitting cannot support: with per-curve parameters there is no held-out prediction because every curve has its own constants. The PINN’s global parameters predict held-out conditions directly, providing an evaluation channel unavailable to the original analysis.

### 4.3 Cross-Comparison with Literature Activation Energies

The growth and dissociation experiments share the same Chen apparatus and use the same *in situ* Raman methodology but probe distinct physical regimes: planar film propagation at the methane-water interface (formation) and homogeneous radial film loss (dissociation). The two PINNs independently recover Arrhenius activation energies:

- Growth: *E*_*a*_ = 58.2 ± 4.7 kJ/mol (LOPO across 6 folds × 5 seeds, *n*_anchor_ = 2.5)
- Dissociation: *E*_*a*_ = 61.4 ± 15.6 kJ/mol (LOCO across 8 folds, fixed *l*)

Both PINNs recover Arrhenius activation energies that can be compared against independent measurements. Reported methane-hydrate activation energies span a wide range—intrinsic dissociation values near 78 to 81 kJ/mol^22,23^ and formation/growth values that are lower and more variable, commonly 40 to 60 kJ/mol^27^—and the recovered values, 58.2 ± 4.7 kJ/mol for growth and 61.4 ± 15.6 kJ/mol for dissociation, both fall within this envelope. The dissociation estimate is only loosely constrained due to the limited Arrhenius leverage offered by the dissociation data set of three pressures across a narrow 0.1 to 0.3 K superheat window; its seed-fold uncertainty of ±15.6 kJ/mol is too large to resolve any difference between the two directions. We therefore report the activation energies only as a consistency check with prior work and draw no mechanistic conclusion about the relationship between the formation and dissociation barriers.

Recovering both activation energies within a single inverse framework is itself an advantage of the PINN over per-condition fitting. In the per-condition regressions of the source works^20,21^ the activation energy is an end-product of an intermediate regression on already-fitted rate constants; the two experiments produced (*E_a_*)_growth_ ≈ 65 kJ/mol and (*E*_*a*_)_dissoc_ ≈ 91 kJ/mol, both within the reported literature range. The per-curve dissociation value is, moreover, sensitive to the film-thickness fits, which can absorb morphological variation. Instead, each PINN recovers a single global activation energy directly, removing that per-curve freedom. A future experiment measuring growth and dissociation under matched (P, ΔT), analyzed with a joint inverse problem, would compare the two activation energies directly.

## 5 Conclusions

Using experimental data from thermoelectrically cooled microreactor systems, this study effectively demonstrates the development and application of PINNs for simulating the dynamics of methane hydrate formation and dissociation. By embedding the governing heat- and mass-transfer balances directly in the loss function and automatically balancing the data and physics residuals through learnable Kendall homoscedastic-uncertainty weights, the framework recovers a single globally consistent set of apparatus-level kinetic parameters across all experimental conditions, in contrast to the per-condition parameter fits of the original analyses.

For the growth data set, the PINN reaches an in-sample velocity RMSE of 3.26 ± 0.17 μm/s, a 23.5% reduction relative to the closed-form serial-resistance model, while constraining the kinetics with seven global physical parameters rather than eighteen per-pressure constants; leave-one-pressure-out cross-validation yields a held-out RMSE of 5.40 ± 0.31 μm/s. For the dissociation data set, the model recovers an intrinsic rate constant *k*_0,ref_ = 0.0612 ± 0.0002 s^–1^ across seeds—within the range of the original per-curve fits—and achieves a leave-one-condition-out held-out RMSE of 0.071. The growth and dissociation activation energies, 58.2 ± 4.7 and 61.4 ± 15.6 kJ/mol respectively, are both consistent with previously reported values within the uncertainty of the fits, which is too large to resolve any difference between the two directions. Beyond predictive accuracy, the inverse-problem formulation makes the identifiability structure explicit, exposing the degenerate (*h*_eff_, *n*_heat_) heat-balance ridge in the growth model and the limited Arrhenius leverage of the dissociation data set rather than concealing them within per-condition parameters.

A key advantage of the PINN framework is its ability to enforce physical consistency even with partial knowledge of the governing equations, making it particularly valuable for systems with limited experimental data or incomplete theoretical understanding. The integration of conductive heat transfer and mixed mass-transfer–crystallization kinetics provides a robust foundation for predicting hydrate behavior across validated operating conditions of 30.0–80.9 bar pressure and 1.0–4.0 K subcooling.

For hydrate-related applications in energy production, carbon sequestration, and climate modeling, this study establishes PINNs as a scalable and efficient computational tool. The demonstrated ability to bridge the gap between traditional physics-based simulations and purely data-driven models opens new avenues for accelerating hydrate research and enabling real-world implementations. Future work should aim to extend the PINN framework to larger-scale systems, mixed-gas hydrates, and real-time operational control scenarios to fully realize the potential of physics-informed machine learning in hydrate science and engineering.

## Data Availability

The simulation codes developed in this study are available from the corresponding authors upon request.

## Abbreviations and Nomenclature

CFD: computational fluid dynamics
IC: initial condition
LOCO: leave-one-condition-out cross-validation
LOOCV: leave-one-out cross-validation
LOPO: leave-one-pressure-out cross-validation
MLP: multilayer perceptron
NLL: negative log-likelihood
NTK: neural tangent kernel
ODE: ordinary differential equation
PDE: partial differential equation
PINN: physics-informed neural network
RMSE: root-mean-square error
sI: structure-I (cubic) methane hydrate
*C*: dissolved-methane concentration field (mol m^–3^)
*C̃*: methane concentration scale, 2500 mol m^–3^
*C*_0_: methane concentration at the front, C­(ξ 0) (mol m^–3^)
*C*_m_: methane concentration at the interface (mol m^–3^)
*C*_w_: water concentration (mol m^–3^)
*D*: methane diffusivity (m^2^ s^–1^)
*d*_β_: pressure exponent of the mass-transport length δ (−)
*E*_a_: Arrhenius activation energy (kJ mol^–1^)
*f*_θ_: raw scalar output of the dissociation network (−)
*h*_eff_: effective heat-transfer coefficient (W m^–2^ K^– n^)
*h*_eff,ref_: effective heat-transfer coefficient at P_ref_ (W m^–2^ K^– n^)
*h*_p_: per-pressure heat-transfer constant, serial-resistance model (W m^–2^ K^– n^)
*h*_α_: pressure exponent of h_eff_ (−)
*k′*: apparent specific crystallization rate constant, k′(T) (m s^–1^)
*k*_b_: crystallization (reformation) rate constant (s^–1^)
*k*_c_: apparent crystallization rate constant in excess water (m s^–1^)
*k*_d_: dissociation rate constant, k_d_(P) (s^–1^)
*k*_m_: mass-transfer coefficient of methane (m s^–1^)
*k*_p_: per-pressure kinetic constant, serial-resistance model
*k*_ref_: Arrhenius rate constant at T_ref_ (growth) (m s^–1^)
*k*_0,ref_: intrinsic dissociation rate constant at T_ref_ (s^–1^)
*K*_eq_: equilibrium constant (−)
*l*: dissociation film thickness (μm)
*M*_CH4_: molar mass of methane (kg mol^–1^)
*n*_d_: moles of dissociated hydrate (mol)
*n*_H_: remaining moles of hydrate (mol)
*n*_0_: initial moles of hydrate (mol)
*n*_anchor_: prior (anchor) value of n_heat_ (−)
*n*_heat_: heat-balance exponent (−)
*P*: pressure (bar)
*P*_ref_: reference pressure, 55 bar (bar)
*R*: universal gas constant (J mol^–1^ K^–1^)
*r*_c_: film-growth rate, mixed mass-transfer–kinetics control (μm s^–1^)
*r*_h_: film-growth rate, conductive heat-transfer control (μm s^–1^)
*T*: temperature field (K)
*T*_b_: bulk temperature (K)
*T*_eq_: phase-equilibrium temperature (K)
*T*_ref_: Arrhenius reference temperature (K)
*T*_0_: front temperature, T­(ξ 0) (K)
*t*: time (s)
*u*_conv_: dimensionless convection coefficient, ρ_H_/ρ_W_ ≈ 0.92 (−)
*v*: front-propagation (crystal-growth) velocity (μm s^–1^)
*x*: neural-network input feature vector (−)
α: heat-transport coupling coefficient, α­(ΔT) (−)
*ΔH*: enthalpy of crystallization/dissociation (kJ kg^–1^)
*ΔT*: subcooling (growth) or superheat (dissociation) (K)
δ: mass-transport (boundary-layer) length (μm)
δ_ref_: mass-transport length at P_ref_ (μm)
η: remaining hydrate mole fraction, n_H_/n_0_ (−)
θ: neural-network and physical parameters (−)
κ: effective thermal conductivity (W m^–1^ K^–1^)
λ_mono_: weight of the monotonicity penalty (−)
λ_n_: weight of the n_heat_ anchor term (−)
ξ: dimensionless front-attached coordinate, ξ ∈ [0, 1] (−)
ρ_H_: hydrate mass density (kg m^–3^)
ρ_W_: water mass density (kg m^–3^)
σ_i_: learnable Kendall noise scale of loss term i (per-term units)
σ_v_: data (measurement) noise scale, growth (μm s^–1^)
σ_data_: data noise scale, dissociation (−)
σ_pde, bc, fmass, fheat, ode_: Kendall noise scales of the physics-residual streams (−)
σ_seed_: within-seed standard deviation across folds (per-parameter units)
σ_fold_: within-fold standard deviation across seeds (per-parameter units)
σ_total_: within-seed and within-fold standard deviations combined in quadrature
τ: time-scale constant in the hard initial-condition ansatz (s)
ω: mass fraction of methane in the hydrate (−)
L: total training loss (−)
L _data_: data-misfit loss (−)
L _pde_: PDE-residual loss, growth (−)
L _bc_: boundary-condition residual loss, growth (−)
L _fmass_: front mass-balance residual loss, growth (−)
L _fheat_: front heat-balance residual loss, growth (−)
L _ode_: ODE-residual loss, dissociation (−)
L _mono_: monotonicity-penalty loss, growth (−)
